# Diet of the wild boar (*Sus scrofa*): implications for management in forest-agricultural and urban environments in South Korea

**DOI:** 10.7717/peerj.7835

**Published:** 2019-10-11

**Authors:** Seong-Min Lee, Eun-Jae Lee

**Affiliations:** 1 Urban Planning Research Division, Daejeon Sejong Research Institute, Daejeon, South Korea; 2 Department of Forest Sciences, College of Agriculture and Life Sciences, Seoul National University, Seoul, South Korea

**Keywords:** Food habit, Human-wildlife conflict, Crop damage, Stomach analysis, Earthworm consumption, Wild boar attack

## Abstract

The wild boar is one of the most widely distributed in the world. In South Korea, the wild boar population has rapidly increased and their habitat use has expanded from forests to urban environments. This expansion has led to increased conflicts with humans, such as the severe damaging of crops and the attacking of people in urban areas. We assessed the stomach contents of wild boar killed by hunters in two different environments in Geochang and Seoul, South Korea, from 2012 to 2017. We compared the feeding habits between sites and between seasons and explored the relationship between the number of earthworms and the main diet. The diet of wild boars inhabiting the two environments were found to differ and vary seasonally. Wild boar in Geochang preferred crops, when available, to natural food resources. Although wild boar in Seoul also preferred crops, they had a higher composition of natural food in their diets because of a low availability of crops. The preference of crops and discarded food waste in urban areas is expected to have accelerated the appearance of wild boar in urban areas. The consumption of earthworms did not differ between the two study sites, but it did differ seasonally due to availability. The number of earthworms was significantly negatively correlated with crop availability in both sites. Effective management plans that involve targeted hunting by baiting with food in Seoul and direct hunting in the fall in Geochang should be implemented to resolve the human–wild boar conflicts in these areas.

## Introduction

Resolving human–wildlife conflicts is one of the main challenges in wildlife conservation and management. In recent decades, many biologists worldwide have focused on ways to resolve these conflicts, which include crop damages, attacks by wildlife, and vehicle collision (Conover, 2001). The wild boar (*Sus scrofa* L.) is one of the most widely distributed in the world and occupies a wide variety of natural habitats (Massei & Genov, 2004). These habitats range from semi-deserts to tropical rain forests, temperate woodlands, grasslands, and reed jungles, as well as anthropogenic environments, and are often found foraging on agricultural land (Schley & Roper, 2003; Massei & Genov, 2004). Wild boars have a high reproductive potential and can rapidly increase their population size, which has led to severe economic damages in many parts of Europe (Keuling, Stier & Roth, 2008). Moreover, wild boar can seriously damage forest ecosystems and transmit diseases to livestock (Bratton, 1975; Reimoser & Gossow, 1996; Brauer, Lange & Kaden, 2006; Barrios-Garcia & Ballari, 2012).

In South Korea, the wild boar was the most frequently poached animal (for illegal gallbladder trade) until the late 1990s (Ministry of Environment, 2010). After the implementation of anti-poaching legislation and the extinction of top predators, such as tigers, leopards, and wolves, in the early and mid-1900s, wild boar populations have been rapidly increasing (National Institute of Biological Resources, 2017; Jo, Baccus & Koprowski, 2018). In addition, wild boars have expanded their range from their native forest habitats to urban areas and some islands. As a result, human–wild boar conflicts have increased in South Korea, and mainly involve severe damages to crops and confrontations between humans and boars in urban areas, such as Seoul (the capital of South Korea). Crop damages by wild boar exceed $5 million per year (about 52% of a total wildlife crop losses), which is the highest among damages caused by wildlife (National Institute of Biological Resources, 2017). Furthermore, in Seoul, wild boar sightings reported by citizens have dramatically increased since 2012 and exceeded 600 cases in 2016. These sightings mainly occur before midnight, from September to November, in apartment parking lots and city parks adjacent to mountains in the downtown areas (Seoul Metropolitan Fire & Disaster Headquarters, 2017). To date, three people have been killed and 22 have been injured by wild boar attacks and automobile collisions throughout the country (National Institute of Biological Resources, 2017).

As an opportunistic omnivore, the wild boar can feed on a wide variety of foods available in a diverse array of habitats. Thus, the diet of wild boars can reflect certain environmental characteristics of the area that they inhabit (Herrero et al., 2006). In particular, food availability is one of the most important factors that can affect the population characteristics of wild boar (e.g., body condition, density, fecundity, juvenile survival rate, and mortality) and individual home ranges (Singer et al., 1981; Groot Bruinderink, Hazebroek & Van Der Voot, 1994; Massei, Genov & Staines, 1996; Geisser & Reyer, 2005; Cellina, 2008; Focardi et al., 2008). Moreover, the activity patterns of wild boar in human-dominated areas are also affected by human activities (Ohashi et al., 2013), and such activities may be the key reasons for the occurrence of wild boar in the urban areas.

Although the diet of wild boar has been extensively documented by many authors, earthworm consumption, one of the main food items of wild boar, has been rarely described owing to the time and physical effort required for the research (Fournier-Chambrillon, Maillard & Fournier, 1995; Baubet, Roper-Coudert & Brandt, 2003; Baubet, Bonenfant & Brandt, 2004). Earthworms play an important role in food chains in temperate ecosystems and are eaten by omnivores and even numerous carnivores (e.g., Eurasian badger (*Meles meles*), red fox (*Vulpes vulpes*), and grizzly bear (*Ursus arctos horribilis*)) (Reynolds & Aebischer, 1991; Mattson, French & French, 2002; Baubet, Roper-Coudert & Brandt, 2003; Cleary et al., 2011). Earthworms also act as intermediate hosts of nematodes that infect wild boar lungs, and thus, infection could occur through consuming earthworms (Humbert & Henry, 1989; Popiolek et al., 2010; Senlik et al., 2011). Earthworms contain high levels of energy and nutrients and consumption of earthworms may influence the growth and mortality of piglets and their sexual maturity in wild boar populations (Henry, 1987; Massei, Genov & Staines, 1996). Thus, quantifying the consumption of earthworms is essential to elucidate the ecology of wild boar.

In many animal species, the young learn which food items are suitable to eat based on what their parents feed them (Mirza & Provenza, 1992). These preferences can have a long-lasting effect on their food selection throughout their life-time and can vary seasonally according to food availability. Numerous studies have assessed the diet of wild boar in many European countries and throughout the USA. However, little is known about the diet of the wild boar in Asia, except for Japan (Asahi, 1995; Kodera et al., 2013). In particular, the diet of wild boars that inhabit urban areas are poorly understood.

Wild boars have a significant impact on plant and animal communities, in their native and introduced ranges. They overturn extensive areas by rooting in search for food, which results in the disturbance of soil cover and plant species composition, and they also compete for foods with native animals (Focardi, Capizzi & Monetti, 2000; see Massei & Genov, 2004; Barrios-Garcia & Ballari, 2012 for review). In addition, natural foods found in the wild boar diet are closely linked to management strategies (i.e., supplementary feeding, baited hunting) (Cellina, 2008; Ballari et al., 2015; Zeman et al., 2018). Knowledge of diet not only contributes to the understanding of energy requirements needed to survive and breed, but also provides insights about interactions between species and their environment (Sih & Christensen, 2001; Cuevas et al., 2013). Therefore, to develop effective management plans to reduce human–wild boar conflicts in agricultural and urban environments and manage wild boar populations, it is important to first understand general foraging habits of wild boar, which can be elucidated by investigating their diets.

The aims of the present study were to: (1) understand the annual and seasonal changes in the diets of wild boars that inhabit forest-agricultural and urban environments in South Korea, (2) quantify earthworm consumption by wild boar between areas. We hypothesized that if wild boars are opportunistic consumers of earthworms, the number of earthworms ingested would vary depending on the availability of earthworms and other main food items, and (3) suggest effective management plans to reduce the human–wild boar conflicts in the specific study areas.

## Materials and Methods

### Study areas

**Figure 1:**
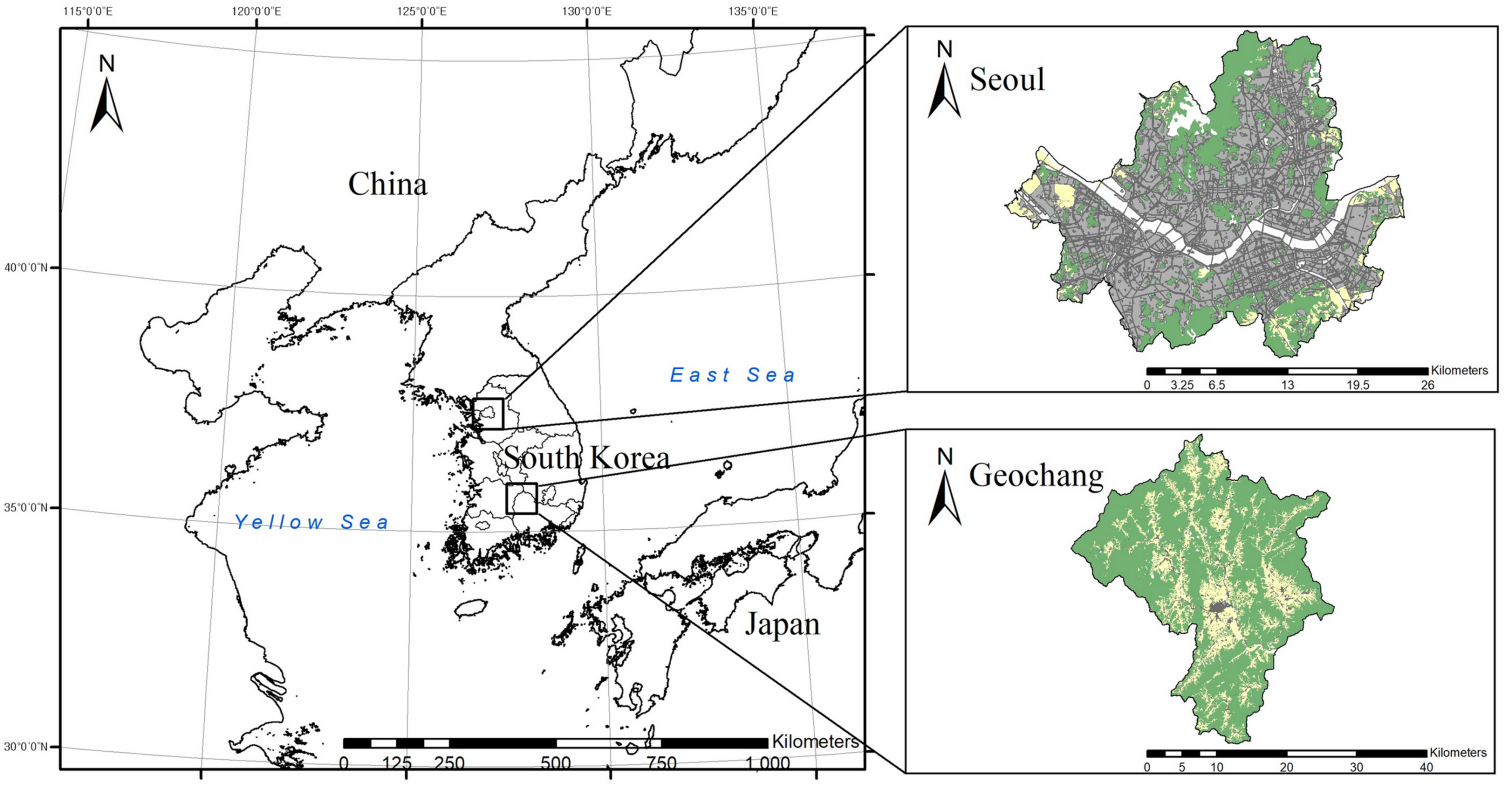
Map of the study areas in South Korea: urban environment (Seoul) and forest-agricultural environment (Geochang). Green: forest; yellow: agricultural land; gray: urban area.

This study was conducted at two different sites in South Korea. The first study site, Geochang (35°41′N, 127°55′E; 803.2 km^2^), is located in the southern part of South Korea (Fig. 1). The landscape of this area is characterized by forest-agricultural land, of which the forest and agricultural land account for 75% and 20% of the total area, respectively. The elevation of this site ranges from 200 to 1,200 m above sea level, and the vegetation is dominated by mixed forests of Sawtooth oak (*Quercus acutissima*), Mongolian oak (*Q. mongolica*), Chinese cork oak (*Q. variabilis*), and Red pine (*Pinus densiflora*). The mean temperature is 12.0 °C and temperatures range from −12.8 to 34.6 °C between winter and summer, respectively. The annual precipitation is 1,242 mm, with the maximum precipitation occurring in August (457 mm) and the minimum occurring in winter (45 mm). The human population density in Geochang (which is representative of the population density in agricultural areas in South Korea) is 79 people/km^2^. The main types of cultivated crops in this area are rice (*Oryza* spp.), apples (*Malus* spp.), and chestnuts (*Castanea crenata*). Chestnut trees are rarely found in the wild and are mostly cultivated as a crop species in the study areas (Geochang County, 2015).

The second site, Seoul (37°41′N, 127°11′E; 605.2 km^2^), is the capital of South Korea and is located in the central part of the Korean Peninsula (Fig. 1). The landscape of this area is mainly comprised of highly developed cities, with the small, fragmented forests only accounting for 25% of the total area. Numerous small house gardens and orchards, which are positioned adjacent to the forests, account for only 0.7% of the total area of Seoul. The vegetation in the area is mainly dominated by deciduous forests of Sawtooth oak and Mongolian oak, and small mixed forests with Red pine and Korean mountain ash (*Sorbus alnifolia*). This study site has the highest human population density (16,861 people/km^2^) in South Korea. The climate and annual precipitation in Seoul are very similar to those in Geochang (Seoul Metropolitan Government, 2017).

In Seoul, wild boars mainly inhabit the forests that surround the cities, and their capturing or hunting is only allowed in emergency situations when wild boar appear in downtown areas. In Geochang, however, the local government allows wild boar to be hunted year-round to control the nuisance caused by boars and to protect crops. At the time of this study, no supplementary feeding had been implemented at either study sites.

### Data collection and analyses

Samples were collected from wild boar that had been killed by hunters at both sites. We analyzed the stomach contents collected from 207 individuals: *n* = 144 in Geochang from 2012 to 2015, and *n* = 63 in Seoul from 2015 to 2017. Stomach contents were stored at −20 °C before analysis. A total of 20% of the mixed stomach contents were sampled and washed over a series of four different sized mesh sieves (5.6 mm, 2.0 mm, 1.0 mm, and 38 μm). The fractions on the 5.6 and 2.0 mm sieves were dried in an incubator for 24 h at 100 °C. The food items were categorized and weighed to an accuracy of ±0.01 g. The setae retained on the 38 μm was used to count the number of earthworms ingested by the wild boars following the method described by Baubet, Roper-Coudert & Brandt (2003). Using the Korean Earthworm Database (2013), we averaged the number of metameres per earthworm of six species (*Amynthas agrestis, A. heteropadus, A. hupeiensis, A. koreanus, Eisenia fetida, E. rosea*) that are commonly found in dry fields and orchards in South Korea (Hong & Kim, 2007; Hong, 2014).

### Statistics

The data are expressed in dry weight (%) and frequency of occurrence in the samples. The composition of each food category (%) was Arcsin transformed for normal distribution. A MANOVA test was used to determine seasonal variation, site differences, and interaction effects. Seasons were defined as: spring (March–May), summer (June–August), fall (September–November), and winter (December–February). Since the sample sizes (i.e., number of sampled stomach contents) differed between the two study sites, a Mann–Whitney *U* and Kruskal–Wallis non-parametric test were conducted to assesses the differences in consumption of earthworms between the two sites. After the Kruskal–Wallis test, a Dunn post hoc test for multiple comparisons was conducted for each site. In addition, we compared the relationship between the number of earthworms and the main food items consumed at each site. Count data often has a Poisson distribution, but the wild boars were often observed to consume only three or four items at a time amongst all listed dietary items. An excessive number of zeros in count data can result in the models being zero-inflated, which could result in the overdispersion of data in the models (Zuur, Savaliev & Ieno, 2012). Therefore, we used a zero-inflated generalized linear model (GLM) with Poisson distribution and a log-link function to explore the relationships. In the event of over- or under-dispersions, we re-fitted the models with a negative binomial distribution. We used log-transformed fresh stomach weights as an offset in the models to deal with the heterogeneity of each sample. Insufficient sample sizes, such as for seeds in Seoul and items that were not the main diet components, such as animal materials, were removed from the models for more robust analyses. All statistical analyses were performed using R 3.5.2 (R Development Core Team, 2011).

## Results

In this study, a total of five main food categories were analyzed from wild boar stomach contents. Plant materials, which included crops, was the main food category, that is, had the highest dry weights (96.6% in Geochang and 91.9% in Seoul) and occurrence frequencies in the diets of the wild boars in both sites.

In Geochang, wild boars consumed more crops than acorns, but conversely, in Seoul, acorns were consumed more frequently than crops (Table 1). Although invertebrates contributed very little to the overall dry weight, their occurrence frequency was as high as 62.5% and 68.3% in Geochang and Seoul, respectively. In terms of their frequency of occurrence, vertebrates represented the smallest proportion of the ingested food categories in Geochang and Seoul (12.5% and 19.0%, respectively). Rice and chestnuts were the most frequently observed food types in the diet of wild boar and the most important food items in the “crops” category in Geochang. However, maize and chestnuts were more frequently observed in the wild boar diet in Seoul. Moreover, in Seoul, wild boars consumed a small amount of food waste in addition to natural food resources (Tables 1 and 2).

**Table 1:** Overall diet composition of wild boar in Geochang (from 2012 to 2015) and in Seoul (from 2015 to 2017) in South Korea, based on the stomach content analyses.

Food item	% of dry weight		% of occurrence	
	Geochang (*n* = 144)	Seoul (*n* = 63)	Geochang (*n* = 144)	Seoul (*n* = 63)
Plant material	53.5	79.2	85.4	93.7
Stems/leaves	19.6	8.2	60.4	49.2
Roots	23.3	20.6	40.3	36.5
Seeds	5.8	3.1	16.7	12.7
Flesh fruits	3.6	12.3	10.4	19.0
Acorns	0.3	34.9	6.3	55.6
Invertebrates	0.9	2.1	62.5	68.3
Insects	0.9	2.1	34.7	42.9
Earthworms	+	+	47.9	58.7
Vertebrates	3.5	3.4	12.5	19.0
Mammals	2.0	1.1	5.6	9.5
Birds	0.8	1.5	2.8	6.3
Amphibians	0.7	0.9	4.9	3.2
Crops	43.1	12.7	55.6	36.5
Apples	9.3	0.0	14.6	0.0
Chestnuts	14.5	4.8	28.5	25.4
Maize	1.1	4.2	2.8	7.9
Potatoes	0.1	0.0	0.7	0.0
Rice	15.3	0.0	22.9	0.0
Sweet potatoes	2.7	3.7	2.1	4.8
Artificial material	0.0	2.7	0.0	14.3
Food waste	0.0	2.7	0.0	14.3

**Note:**

+, trace amounts.

**Table 2:** Seasonal variations in the main diet compositions of wild boar from two sites in South Korea: Geochang (from 2012 to 2015) and Seoul (from 2015 to 2017).

Food item consumption (g)	Spring		Summer		Fall		Winter	
	Geochang (*n* = 23)	Seoul (*n* = 9)	Geochang (*n* = 58)	Seoul (*n* = 9)	Geochang (*n* = 39)	Seoul (*n* = 37)	Geochang (*n* = 24)	Seoul (*n* = 8)
Plant material	97.8	94.1	51.8	95.5	21.9	71.9	77.7	82.1
Stems-leaves	31.7	3.4	28.7	15.0	11.9	12.4	30.9	11.9
Roots	57.6	39.4	11.0	11.9	4.4	11.3	42.3	62.4
Seeds	8.0	0.0	1.1	6.3	3.0	4.7	0.9	0.1
Fruit flesh	0.0	0.0	11.0	51.9	2.4	2.5	0.2	0.0
Acorns	0.4	51.3	0.0	0.4	0.2	41.0	3.4	7.8
Animal material	1.0	5.9	4.1	1.4	5.3	8.1	3.8	13.1
Crops	1.2	0.0	44.1	13.1	72.7	14.7	18.5	0.0
Apples	1.2	0.0	17.6	0.0	4.3	0.0	4.6	0.0
Chestnuts	0.0	0.0	0.8	1.4	47.1	9.1	7.0	0.0
Maize	0.0	0.0	4.1	11.7	0.0	1.4	0.0	0.0
Rice	0.0	0.0	16.6	0.0	21.3	0.0	6.8	0.0
Sweet potatoes	0.0	0.0	4.3	0.0	0.0	4.3	0.0	0.0
Artificial material	0.0	0.0	0.0	0.0	0.0	5.3	0.0	4.8
Food waste	0.0	0.0	0.0	0.0	0.0	5.3	0.0	4.8

The predominant dietary items during all seasons in both habitats were vegetable parts, but the proportion of these food items in the diet of wild boars in Geochang decreased in summer and fall with the increase in crop consumption. In fall, the wild boars were found to consume the lowest amount of plant materials and the highest amount of crops in Geochang, while, in Seoul, the consumption of plant materials remained relatively high in all seasons. Roots were the main food items during winter and spring in both sites. Acorns were consumed ten times more in Seoul than Geochang in fall and spring when acorn consumption was highest. Fleshy fruits did not constitute a main food item in Geochang in any season, but they were a dominant item in summer in Seoul. In Geochang, wild boars mainly consumed crops from summer (rice and apples) to fall (rice and chestnuts), making up 72.7% of the diet composition. In Seoul, chestnuts were the only crop item that comprised a relatively high proportion of the diet. Animal materials in the diet were consumed frequently and consistently but did not constitute an important food item in either site during all seasons (Table 2).

**Figure 2:**
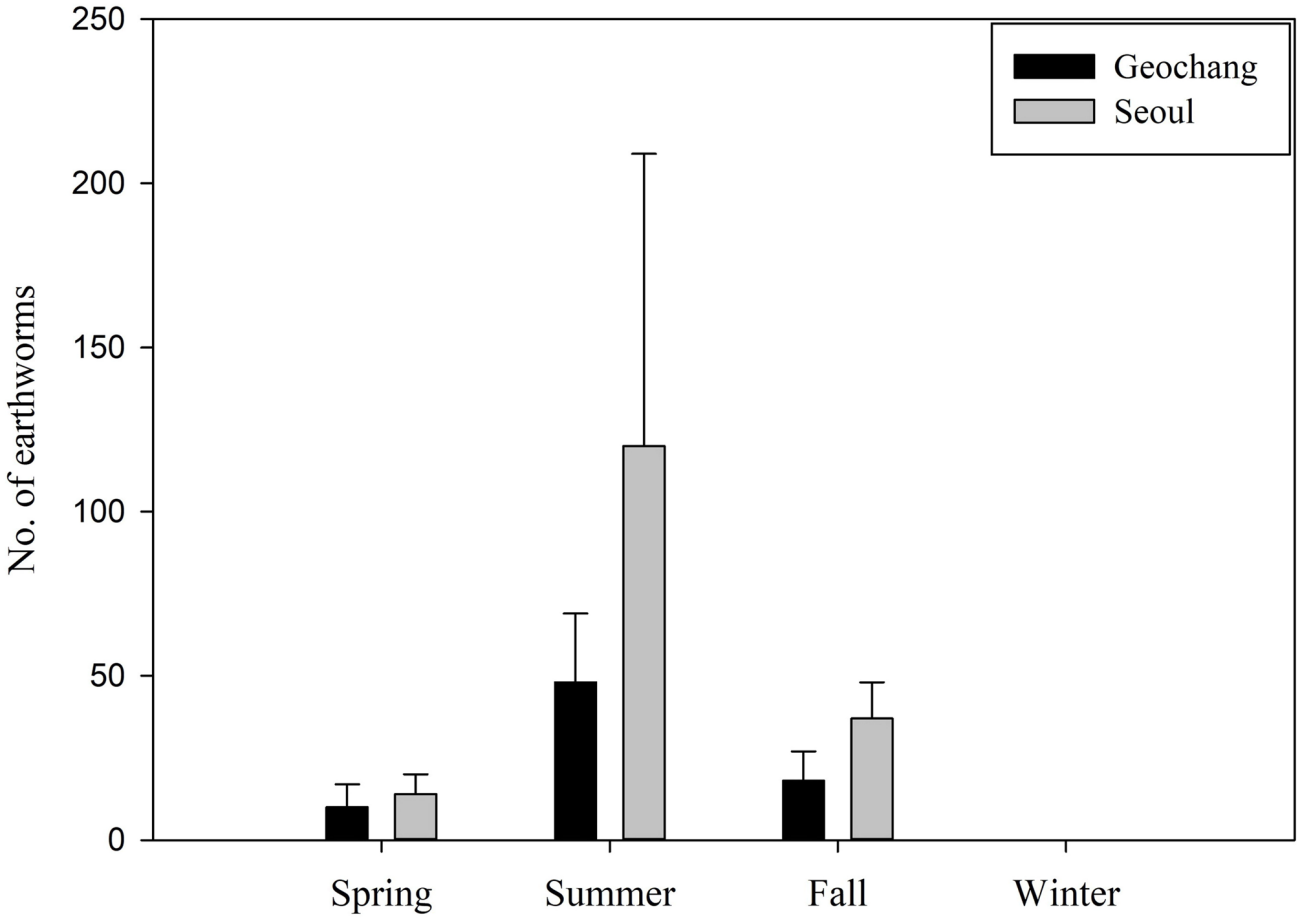
Seasonal variation in the number of earthworms ingested by wild boars in two sites in South Korea. Bars represent means (*n* = 144 in Geochang from 2012 to 2015, *n* = 63 in Seoul from 2015 to 2017) and vertical error bars represent standard errors.

The MANOVA results showed that there were significant differences between sites and between seasons. We also found significant interaction effects between the sites and seasons, which indicates that the differences in site factors were generally dependent on seasonal factors (Table 3). Moreover, there were also seasonal differences in the earthworm consumption in Geochang (Kruskal–Wallis test, χ^2^ = 29.57, *P* < 0.001) and in Seoul (χ^2^ = 9.84, *P* = 0.019). During summer, a significantly higher amount of earthworms was consumed than in spring and fall, but the consumption did not differ between these latter seasons. No earthworms were consumed during winter in either site (Fig. 2). Although the mean number of earthworms ingested by wild boars was higher in Seoul, it was not significantly different from the consumption in Geochang (Mann–Whitney *U*-test, *W* = 3960.5, *P* = 0.119). The results of the zero-inflated GLMs revealed that crops and acorns in Geochang, and only crops in Seoul were significantly negatively correlated with the number of earthworms ingested by wild boars. Wild boars tended to ingest fewer earthworms when they consumed a larger amount of crops (Table 4).

**Table 3:** The summary of the MANOVA results for wild boar comparisons between seasons and between two sites in South Korea.

Effect	Wilks	*F*	d*f*	*P*-value
Site	0.48	23.01	1, 9	<0.001
Season	0.42	7.13	3, 27	<0.001
Site × Season	0.60	3.95	3, 27	<0.001

**Table 4:** A comparison of the relationships (from zero inflated GLMs) between the main diet and the number of earthworms ingested by wild boar inhabiting two sites in South Korea.

Site	Variable	Estimate	SE	*Z*	*P*-value	
Geochang	Intercept	−2.707	0.316	−8.575	<0.001	
	Stems/leaves	−0.031	0.043	−0.718	0.473	
	Roots	−0.030	0.045	−0.665	0.506	
	Seeds	−0.026	0.028	−0.814	0.416	
	Flesh fruits	−0.049	0.058	−0.856	0.392	
	Acorns	−0.081	0.016	−2.170	0.030	*
	Crops	−1.605	0.740	−4.998	<0.001	**
Seoul	Intercept	−3.344	0.549	−6.089	<0.001	
	Stems/leaves	0.037	0.118	0.312	0.754	
	Roots	−0.078	0.088	−0.888	0.375	
	Flesh fruits	−0.017	0.034	−0.489	0.625	
	Acorns	−0.050	0.038	−1.341	0.180	
	Crops	−0.183	0.054	−3.400	<0.001	**
	Food waste	−0.340	0.310	−1.228	0.220	

**Notes:**

* *P* < 0.05.

** *P* < 0.001.

## Discussion

The wild boar is an opportunistic omnivorous species with a diet that is mainly based on plant material, including crops, in forest-agricultural lands and urban areas. Wild boars prefer crops to natural food resources, during the harvest season, regardless of the habitat type (Fournier-Chambrillon, Maillard & Fournier, 1995; Baubet, Bonenfant & Brandt, 2004; Herrero et al., 2006; Cellina, 2008; Merta et al., 2014). Maize and sweet-potatoes are crops that are known to be preferred by wild boars. However, in Geochang, apples, chestnuts, and rice were the main crops that were consumed by the wild boar in summer and fall. These crops are not only widely cultivated, but the farmlands are also adjacent to forest edges. These conditions increase food availability for wild boar. Environmental variables, such as the distance to forests and streams, and the forest edge shape can affect crop damage by wild boar (Thurfjell et al., 2009; Lee et al., 2018). Ballari & Barrios-Garcia (2014) also documented that food availability and energy requirements were important factors that determined the diet of wild boars. Our findings suggest that crop availability was more influential than crop species, which is informative for developing management strategies for wild boar populations, such as trapping, baited hunting, and dog hunting in South Korea.

In Geochang, wild boars consumed crops to a greater extent than natural food items in the fall. Even though oak trees were predominant in this area (National Institute of Ecology, 2014), wild boar preferentially consumed chestnuts over acorns throughout the study period. Even in winter, wild boar consumed remnants of the crops left in the fields after harvesting. However, these findings could be related to a mast-failure year (Massei, Genov & Staines, 1996; Herrero et al., 2005), but these events depend on the aspect, elevation, and region even within the same mountain habitat area in South Korea (Kim et al., 2012). On the other hand, in Seoul, the wild boar consumed acorns to a similar degree as reported in other studies when the availability of chestnuts was low (Genov, 1981; Henry & Conley, 1972; Groot Bruinderink, Hazebroek & Van Der Voot, 1994). Chestnuts are bigger in size and thus their consumption (per unit) would result in a more rapid intake of a larger quantity of nutrients relative to the intake of acorns. In addition, chestnuts contain relatively lower tannin contents than acorns (Yook, Lee & Kim, 2002). Tannins reduce an animal’s ability to absorb proteins and carbohydrates by binding to them, and as a result, important nutrients are excreted rather than absorbed (Robbins, 1983). Thus, the wild boars inhabiting areas where they can consume chestnuts were likely to prefer them over acorns. Wild boars inhabiting Seoul came from the habitats surrounding the city, thus they might have also maintained the habit of preferring chestnuts and rice over natural food sources if crops are available even though they inhabit urban areas; especially in the fall, which is a very important season in preparation for the winter. However, the wild boar may consume acorns in the fall if they fail to locate other energy-rich food sources in order to prepare for the winter in urban areas like Seoul.

Singer et al. (1981) reported that the home range of the wild boar increased when food availability was insufficient. Massei, Genov & Staines (1996) demonstrated a strong dependence on energy-rich food in their diet throughout the range, irrespective of the habitat and latitude. Some wild boars may, therefore, roam around the cities searching for higher energy food sources, such as crops, outside of their mountainous habitats. This may explain why the highest number of wild boar sightings in urban areas was reported by citizens in the fall, which accounts for 45.7% (621 cases) of the total sightings (1,363 cases) reported from 2012 to 2016 in Seoul (Seoul Metropolitan Fire & Disaster Headquarters, 2017). Wild boar may also consume food waste as an energy-rich food during their roaming throughout urban areas (Table 2). In cities, some residents discarded food waste in small orchard gardens as compost or illegally in secluded areas. Food waste discarded close to forests (i.e., wild boar habitat) might also be contributing to the increase in urban visits by attracting the wild boar to the urban areas. In human-dominated areas like Seoul, wild boars are usually more active during the night-time because of human disturbance during the daylight hours (Ohashi et al., 2013). Wild boars may initiate searches for preferred food sources in the early evening, which may also explain why most of the reported sightings of wild boar occurred before midnight (Seoul Metropolitan Fire & Disaster Headquarters, 2017). Our results indicate that food preferences as well as energy requirements of wild boar at different periods could be important to consider to reduce wild boar–human conflicts in urban areas.

In the present study, roots were the most frequently observed stomach contents in winter. This phenomenon was probably related to a low availability of other items during winter or because the boars sought food sources to balance their nutrition requirements. Schley & Roper (2003) documented that wild boar that obtained their caloric requirements from crops would need to compensate for the lack of dietary protein by consuming root-like foods. Natural habitats, such as Geochang, have a high diversity of species and a complex forest structure. In such habitats, wild boars are able to consume roots throughout the winter until spring. However, urban forests are less complex and do not contain enough root resources to sustain the wild boar until spring. As a result, the wild boar in urban areas compensated their diet by consuming remnant acorns again in early spring (Table 2). In our stomach content analysis, wild boar in South Korea would tend to consume acorns when crops were unavailable in fall or availability of other food items were low in early spring.

Wild boars ingested earthworms throughout the year in both study sites except during winter. Earthworm consumption is likely to be related to climatic variables, especially precipitation. Such variables cause earthworms to rise to the surface layer of the soil, and thus increase their availability for consumption by wild boars (Fournier-Chambrillon, Maillard & Fournier, 1995; Baubet, Bonenfant & Brandt, 2004). Precipitation is strongly concentrated in the summer in South Korea, and this pattern matches that of the consumption of earthworms by wild boars in our study sites. During winter, earthworms dig deeper into the ground and remain inactive because of the prevalent cold temperatures (Baubet, Bonenfant & Brandt, 2004; Ballari & Barrios-Garcia, 2014), therefore, they are less accessible for consumption. Baubet, Bonenfant & Brandt (2004) demonstrated that the consumption of earthworms by wild boars was negatively related to root consumption. However, in the present study, no earthworms were found during winter when the wild boars heavily relied on roots in their diet and our zero-inflated GLM results showed that earthworm consumption was negatively related to crop consumption. Therefore, we assumed that the wild boars ingested earthworms simultaneously with the natural food through rooting activities. When they consumed crops, they simply picked up food items off the ground surface, especially in ripening season, or separated out the rice using their teeth, rather than using rooting activities; hence they ingested earthworms fewer. Although acorns were also negatively correlated with the consumption of earthworms in Geochang, it is likely due to wild boars having consumed relatively small amounts of acorns with the crops. On the other hand, in Seoul, there was no negative correlation between acorns and earthworm consumption. Wild boar obtain their energy requirements for wintering by consuming both of them since there were less crops than agricultural land. Consumption patterns were similar between the two sites and different between seasons. It seems that wild boars in South Korea are opportunistic predators of earthworms rather than primary (Baubet, Roper-Coudert & Brandt, 2003). Our results also suggested that wild boar might have been infected by lung nematodes from earthworms, which would have led to indirect consequences for their populations in both study sites (Humbert & Henry, 1989; Popiolek et al., 2010; Senlik et al., 2011).

Several authors have emphasized the possibility that animal materials are underestimated in diets because of their high digestibility in scat analysis. In our stomach content analysis, animal matters were consistently consumed by wild boar from spring to fall and exceeded 60% in frequency of occurrence. Thus, animal matters constitute a staple energy source in the diet of wild boars in our study sites. However, our findings were consistent with those of previous studies that showed that vertebrates were not likely to be an important food source for wild boar (Wood & Roark, 1980; Howe, Singer & Ackerman, 1981; Cuevas et al., 2010). Vertebrates, which were found in low quantities, were mostly from carrion scavenging behaviors. Wilcox & Van Vuren (2009) showed that there was correlation between vertebrate predation and body condition. We did not observe this as frequently. Consequently, wild boar in our study area show temporary scavenging behavior when other resources were scarce, particularly in winter.

Our findings indicated that wild boar preferred crops over natural food sources in agricultural environments and they may maintain the habit of searching for high-energy foods such as crops to meet their energy demands in urban environments. Hunting with hounds may help to reduce the population densities of wild boar; however, because the natural habitats (i.e., forests) in Seoul are relatively small compared to those in the agricultural areas or other forested areas, such activities may disturb the wild boar and cause them to flee into urban areas. Seoul is densely populated, and many hikers frequent the areas around the mountains that wild boars inhabit. Consequently, wild boars may accidentally attack people (e.g., bystanders and hikers) out of fear or in defense, especially when being hunted. Therefore, targeted hunting by baiting with crop-based food sources (i.e., their preferred food sources) in fall could serve as an effective and safe management plan to reduce the population density of wild boars in urban areas. However, in forest-agricultural lands, wild boars repeatedly damage cultivated crop fields (Lee et al., 2018). Geisser & Reyer (2004) reported that hunting and electric fences were effective in preventing crop damage. Therefore, the use of electric fences and traps around their preferred crop fields (i.e., apples, chestnuts, and rice) and hunting with hounds (especially in fall) should reduce the population density of wild boars in agricultural areas.

## Conclusions

In this study, a total of five main food categories were analyzed from the stomach contents of wild boar in South Korea. Plant materials comprised the main components and invertebrates contributed very little to the overall dry weight, but their occurrence frequency was high in the diet of wild boar. The diet composition differed between forest-agricultural and urban environments, as well as between seasons. In Geochang, wild boar preferred crops to natural food resources in summer and fall. Apples, chestnuts, and rice were the most preferred crops. These food preferences are likely to have resulted in the wild boar appearing in downtown areas in Seoul, that is, wild boars searching for their preferred food sources. Moreover, food waste discarded close to the mountains and the natural habitats of wild boar may have attracted and accelerated the increase in the occurrence of wild boar in urban areas. Consumption of earthworms by wild boars did not differ between the two sites but did differ significantly among seasons. In addition, a negative relationship was observed between earthworm and crop consumption in both sites.

The findings of the present study suggest that targeted hunting with bait in urban areas and hunting and the installation of electric fences around preferred crops in the fall in agricultural lands would be effective measures to reduce wild boar density and consequently human–wild boar conflicts in South Korea. Further studies are needed to better understand the feeding habits of wild boar, and to enhance the effective management plans addressing damage control and population management.

## Supplemental Information

10.7717/peerj.7835/supp-1 Supplemental Information 1 Raw data of wild boar in two sites, South Korea.

## Additional Information and Declarations

### Field Study Permissions

The following information was supplied relating to field study approvals (i.e., approving body and any reference numbers): All hunters who provided stomach samples for this research got the hunter license and they all got full permission for hunting wild boars from Geochang County and Seoul Metropolitan Government.

### Data Availability

The following information was supplied regarding data availability: The raw measurements are available as a Supplemental File.
